# Dr. Welch’s Article on Smallpox

**Published:** 1901-02

**Authors:** W. F. Blunt

**Affiliations:** State Health Officer; Austin, Texas


### Dr. Welch's Article on Smallpox.

Austin, Texas, Feb. 10, 1901.

*To the Editor of the Texas Medical Journal:*

Owing to the prevalence of a somewhat unusual type of smallpox in many parts of this State at the present time, and for two years past, and the fact that many errors of diagnosis have been made which have been largely responsible for the spread of the disease, I would be much gratified if you would reproduce the illustrated article upon this type of smallpox written by Prof. W. M. Welch, M. D., surgeon in charge of the Municipal Hospital of Philadelphia, (and published by you last February). He is confessedly *the* authority on this subject in the United States. The disease is usually mistaken for chickenpox, impetigo contagiosa, or "Cuban itch". The occurrence of a mortality of from 3 per cent. to 5 per cent. should absolutely exclude either one of the three diseases. As to the latter, "Cuban itch", it is needless to state to any well informed physician that no such disease exists, or is described in medical literature. Owing to the infrequency of smallpox in the past, and the very large number of our physicians who have never seen a case of the disease, or at most a very few cases and those of a malignant type, it might have been expected that many mistakes of the kind would be made at the beginning. But it would seem that by this time the profession of the State should have acquired sufficient knowledge of the present epidemic to enable them to make a correct diagnosis. Dr. Welch calls attention to the danger that this heretofore mild disease may acquire malignancy at any time, which, indeed, it has already done in a number of localities since the time that Dr. Welch wrote the article. Indeed, the mild cases of the disease in any locality of the State is liable to, and in fact usually does, convey to some person exposed to them, a severe and even fatal case of the disease.

The symptoms which should decide the diagnosis are: The preeruptive fever of more than twenty-four hours; the immediate abatement of the fever on the appearance of the eruption; the firm, shot-like sensation conveyed to the fingers by the eruption; the continuance of the eruption for more than four or five days. No weight should be given to the absence of the so-called smallpox odor, or to the absence of the secondary suppurative fever. These symptoms occur in many cases, yet in many others they are absent. I hold that it is the duty of every physician of the State, who has a case of acute eruptive disease, not positively diagnosed as measles or scarlatina, to deal with the same as suspicious of smallpox, and to report as "suspicious" to his county health officer, or to the State health officer at once.

W. F. Blunt,
State Health Officer.

